# Research on aging during the Venezuelan humanitarian crisis: the experience of the Maracaibo aging study

**DOI:** 10.1186/s12889-021-10526-0

**Published:** 2021-03-09

**Authors:** Gladys E. Maestre, Rosa V. Pirela, Carmen L. Paz, Jesus D. Melgarejo, Luis J. Mena, Carlos A. Chavez, Reinier Leendertz, Michele Petitto, Eglé Silva, Gustavo E. Calmón, Lama Al-Aswad, Joseph H. Lee, Joseph D. Terwilliger

**Affiliations:** 1 Department of Neuroscience, University of Texas Rio Grande Valley School of Medicine, One West University Blvd, BROBL, Rm. 106, Brownsville, TX 78520 USA; 2 Department of Human Genetics, University of Texas Rio Grande Valley School of Medicine, Brownsville, TX USA; 3 Laboratory of Neurosciences, University of Zulia, Maracaibo, Venezuela; 4 Universidad Politécnica de Sinaloa, Mazatlán, Mexico; 5 Maracaibo Eye Clinic, Maracaibo, Venezuela; 6 Cardiovascular Institute (IECLUZ), University of Zulia, Maracaibo, Zulia Venezuela; 7 Department of Ophthalmology, Grossman School of Medicine, New York University, New York, NY USA; 8 Sergievsky Center, College of Physicians and Surgeons, Columbia University, New York, NY USA; 9 Taub Institute for Research on Alzheimer’s Disease & the Aging Brain, College of Physicians and Surgeons, Columbia University, New York, NY USA; 10 Department of Neurology, College of Physicians and Surgeons, Columbia University, New York, NY USA; 11 Department of Epidemiology, School of Public Health, Columbia University, New York, NY USA; 12 Departments of Psychiatry and Genetics & Development, Columbia University Medical Center, New York, NY USA; 13 Division of Medical Genetics, New York State Psychiatric Institute, New York, NY USA

**Keywords:** Aging, Dementia, Venezuela, Humanitarian crisis, Research, Ethical challenges, Alzheimer’s, Low-and-middle income countries, Vulnerable populations, Elderly

## Abstract

**Background:**

Venezuela is in the throes of a complex humanitarian crisis that is one of the worst in decades to impact any country outside of wartime. This case analysis describes the challenges faced by the ongoing Maracaibo Aging Study (MAS) during the deteriorating conditions in Venezuela. When the MAS began in 1997, it focused on memory-related disorders. Since then, strategic planning and proactive community participation allowed us to anticipate and address logistical, funding, and ethical challenges, and facilitated the enrollment and retention of more than 2500 subjects over 55 years of age. All participants, who are residents of the city of Maracaibo, Venezuela, underwent various assessments on several occasions. Here, we discuss how our approach to implementing a longitudinal, population-based study of age-related conditions has allowed our research program to continue throughout this period of political, economic, and social upheaval.

**Discussion:**

As the social context in Venezuela became more complicated, new challenges emerged, and strategies to sustain the study and participation were refined. We identified five main mechanisms through which the evolving humanitarian crisis has affected implementation of the MAS: 1) community dynamics; 2) morale of researchers, staff, and participants; 3) financial feasibility; 4) components of the research process; and 5) impact on the health of staff, participants, and their families. Strategies to compensate for the impact on these components were implemented, based on inputs from community members and staff. Improved communication, greater involvement of stakeholders, broadening the scope of the project, and strengthening international collaboration have been the most useful strategies. Particular demands emerged, related to the increased mortality and comorbidities of participants and staff, and deterioration of basic services and safety.

**Conclusion:**

Although the MAS has faced numerous obstacles, it has been possible to continue a longitudinal research project throughout the humanitarian crisis, because our research team has engaged the community deeply and developed a sense of mutual commitment, and also because our project has provided funding to help keep researchers employed, somewhat attenuating the brain drain.

## Background

Large-scale longitudinal biomedical research studies are difficult to conduct under the best of conditions, in the most developed countries of the world. Such studies are critical to our understanding of the age-related processes which affect the development and progression of human disease over the lifespan. It is critical to perform community-based longitudinal studies in the developing world as well, where the environment, lifestyle, and access to healthcare differ in profound ways from wealthy western countries, especially so for elderly individuals. However, social and economic upheaval and humanitarian crises are not uncommon in the developing world, and it can be difficult to maintain an active longitudinal research project during times of economic and political instability. While every instance of social upheaval and economic chaos will be different, there are lessons that can be learned from the experience of researchers who have successfully managed to keep their research going throughout ongoing humanitarian crises.

The aim of this case analysis is to discuss the strategies implemented by researchers of the Maracaibo Aging Study (MAS) to sustain research efforts during the humanitarian crisis in Venezuela. This report is not focused on research findings from the MAS, but on how different procedures for conducting scientific research with older adults were affected by the challenging and changing conditions in Venezuela, and about the different strategies implemented to mitigate the effects of the crisis on the research program.

### Humanitarian context

Venezuela sits on top of 20% of the world’s oil reserves and has long been one of the world’s leading oil exporters. By the turn of the century, when oil prices were skyrocketing, the government had a huge surplus of cash, which they invested in providing social services to its largely impoverished population. The government made food, housing, and healthcare widely accessible to the people. While oil prices were still high, they began revising the constitutional framework to advance what was referred to as twenty-first century socialism, hoping to share the wealth of the nation with the nation’s impoverished masses. Manufacturing, agriculture, mining and the like were taken over by the state, while economic dependency on profits from selling their oil abroad increased dramatically.

This was all fine as long as oil exports remained profitable, but in 2014, global oil prices began to plummet for a variety of external geopolitical reasons. Lacking this all-important source of income, manufacturing and service industries ground to a halt, and the currency collapsed. The resulting hyperinflation led to shortages of electricity, food, and medicine, and the government could no longer afford to pay for the social services it had been providing to the people. This led to social chaos, riots and widespread urban violence, such that today Venezuela has one of the highest rates of violent crime and murder in the world, leading to a mass exodus of more than four million people and creating a refugee crisis in neighboring states. By all rights, Venezuela should be one of the wealthiest countries in the hemisphere, given its natural beauty, resources, and geographical location, but dependency on a single economic sector in its planned economy has made it one of the poorest countries in the Americas.

This economic crisis has been described as among the worst to impact any nation during peacetime in decades [1]. By 2018, more than 90% of the country’s population were living below the poverty line [2]. Families could no longer meet basic needs, and by 2019, an average family could only afford about four-day supply of food per month [3]. Over five million people (17% of the population) left the country between 2018 and October 2020 [4] in the largest mass exodus in modern Latin American history [5]. General public health has been negatively impacted by the lack of medical supplies and pharmaceuticals, the excessive cost of available medicines, and the mass exodus of healthcare personnel, as those with the means fled the crisis [3, 6]. The healthcare system has also been severely imperiled by frequent electrical blackouts: For example, between November 2018 and February 2019, 79 inpatient deaths were directly attributed to a paucity of electricity in operating rooms or intensive care units [7]. There was a dramatic increase in infectious diseases, including tuberculosis and malaria [8], as well as chronic disorders, such as diabetes and cardiovascular disease [9]. The crisis also affected mental health at all levels: The suicide rate increased 2.5 fold between 2015 and 2018 [10]. The burden of these diseases on the population is compounded by the scarcity of food. When resources are thin, older adults have a high risk of being excluded from food distribution in favor of children and working members of the family. The paucity of food and supplies is even more extreme in the State of Zulia where Maracaibo is located, than in central Venezuela, even though Zulia was the most prosperous state in the country prior to 2014.

### Research approach and study design of the Maracaibo aging study

The MAS, initiated in 1997, is a population-based longitudinal study of age-related disease, particularly memory disorders, which has been expanded to include cardiovascular, neurological, and other age-related outcomes. The general aim of the MAS is to describe and analyze the primary cognitive, cardiovascular, nutritional, and social determinants of aging in the local population. The baseline cohort included 2453 subjects residing in downtown Maracaibo, who were then over the age of 55 years. In 2011, an extended family of over 500 individuals from the nearby village of Santa Rosa de Agua was added to the MAS to assess white matter hyperintensities, a surrogate marker for small vessel disease that can be measured by neuroimaging.

The details of the study protocols have been described elsewhere [11]. Briefly, a door-to-door survey was conducted to build a registry of all subjects 55 years or older, living in the target area. The initial sampling frame of the study was the Santa Lucia neighborhood, one of the 18 well-defined areas into which Maracaibo is divided for administrative purposes. This setting provided several advantages for an epidemiological study, such as a high density of houses per block. As it is one of the oldest areas of Maracaibo, a significant number of households with at least one older adult was expected. All subjects were included in a registry after giving informed consent. Every subject was invited to participate in the clinical, neuropsychological, and cardiovascular assessments.

A trained social worker visited the home of each subject and conducted a family interview. An informant (usually a spouse or adult child residing in the same home) knowledgeable about the participant’s daily activities and health issues, was identified and invited to confirm health details and medical history. Neuropsychiatric evaluations were performed by trained neurologists, psychiatrists, or internists, and neuropsychological testing was administered by psychologists. Routine laboratory tests were conducted, and blood was drawn and stored for future genetic analysis. Finally, participants received the results of the evaluations after a clinical consensus conference by the multidisciplinary team of the MAS. To assess age-related changes, the family interview and clinical assessment were repeated every three years in most cases. A community health worker was assigned to the hospital catchment areas to document fatal and non-fatal events among participants. Brain magnetic resonance imaging (MRI) and comprehensive ophthalmological assessment were performed in approximately 500 MAS participants during 2013 to 2016. In 2015, the study added an in-depth ophthalmological examination, including automated visual field testing and optical coherence tomography (OCT) to assess posterior segment pathologies.

### Contributions of the Maracaibo aging study to science

The MAS demonstrated a high prevalence and incidence of Alzheimer’s disease and age-associated dementias in the Santa Lucía cohort [12–14]. The study also measured non-traditional cardiovascular risk factors that were potentially relevant to dementia, such as plasma homocysteine levels [15, 16]. Because high blood pressure is a known risk factor for dementia, including Alzheimer’s disease, the MAS reported the prevalence, treatment, and control rates of hypertension, as well as circadian dysregulations in blood pressure, and ultimately developed a novel index of blood pressure variability [17]. Data from ten populations across the world provided evidence that social, economic, and education factors influence rates of preventable diseases [18]. Of those populations, the MAS reported the second lowest human development index and the highest prevalence of hypertension. The MAS demonstrated the urgent need for capacity building in low resource settings to diagnose dementia and address the problems of affected individuals and their caregivers [19, 20]. Due to the social accountability, community engagement, and capacity-building approaches of the MAS, many community resources have been established over the years, including a School for Non-Professional Caregivers of Older Adults [21], and Workshops for Social, Physical and Cognitive Stimulation for people living with dementia, with a publicly available manual in Spanish [22].

### Methods to understand the impact and response to the humanitarian crisis

For this report, a qualitative approach was used for data collection and analysis to address two specific questions:

- What challenges have the MAS faced since the humanitarian crisis in Venezuela?
- What strategies have the MAS implemented to overcome these challenges?

Collection of information from researchers, staff, and community members was facilitated by listening sessions, group or one-on-one discussions that took place in person, via teleconference, or through emails. In addition to the notes of these sessions, records and laboratory notebooks were reviewed for relevant information.

## Discussion

The MAS was initiated in 1997, during a time when Venezuela was relatively prosperous, especially Zulia State, with most of Venezuela’s oil and cattle resources originating in that region. The Venezuelan crisis evolved slowly over the subsequent 20 years, throughout which time the MAS continued, largely unabated by the gradual social and economic collapse. The data, collected for more than 20 years, increased our understanding of dementia and other prevalent age-related disorders in Hispanic populations. While the MAS was not designed within the context of a humanitarian crisis, today it is one of the few longitudinal studies of the elderly that can provide information about the effects of social and economic collapse.

### Challenges to research

During the planning stage of the MAS, there was a high degree of resistance among local academics about initiating a longitudinal study among the elderly. In a country where, at that time, about 20% of children < 5 years old had low height for their age [23], research benefitting the elderly was perceived as frivolous, irrelevant, or unjustified, compared to other target populations, including children with developmental disabilities or people with hereditary diseases, such as Huntington’s disease. There were no existing aging studies in Venezuela, and age-related disorders were mostly considered to be little more than a normal and unavoidable part of the aging process. The MAS focus on aging was justified extensively by its results prior the humanitarian crisis, which helped the research team argue for the need to address knowledge gaps during the more troublesome times. However, a number of challenges for the MAS have arisen during the humanitarian crisis. We have summarized those as follows.

#### Ethical issues

Increased mortality/morbidity in participants, staff members, and their families has dramatically impacted our study during the humanitarian crisis. The call for participation in the study under deteriorating circumstances has been perceived by some staff members as an additional burden for participants. Even though we made it clear to the participants that there would be no direct health benefit or compensation for participation, some subjects mistakenly inferred that there could nevertheless be potential personal benefits of participation in the study, phenomena called therapeutic misconception and misestimation [24, 25]. Many considered the study to be a potential source of the medical help that they cannot otherwise access, due to the deteriorating public health system and the high cost of private health care [26]. This problem worsened when the study team applied the exclusion criteria established by the research protocol, which mandates that the sickest people be excluded from the study. It has been difficult to deny individuals who are willing to participate, especially when they need the clinical assessments we are conducting.

#### Deterioration of basic services, decreasing safety, and supply problems

The prevalence of poverty in Venezuela has escalated rapidly during the past decade, most dramatically after 2017 [27]. Shortages of food, medicine, electricity, and potable water combined with severe degradation of the sanitation infrastructure has made it difficult to carry out research effectively. Each individual in society must allocate a significant amount of time, money, effort, and patience to obtaining their own basic life necessities. These difficulties have been exacerbated by increasing crime rates, resulting in participants being afraid to wear valuable ambulatory blood pressure monitors in public, and social workers preferring not to use expensive electronic tablets for questionnaires.

Difficulties in obtaining medical and laboratory supplies, as well as spare parts or maintenance for medical equipment, contributed to delays in our timeline. Brain MRIs were repeatedly delayed and rescheduled, due to a scarcity of helium and to problems in obtaining supplies and parts for the equipment.

#### Logistics affecting staff and participants, and staff emigration

Fuel shortages and the increasing lack of public and private transportation services led to difficulties in accessing the study population, especially in Santa Rosa, which is not in the city center. The restricted mobility of staff, participants, and materials/equipment interfered with research activities, and made it impossible for some subjects to participate. Unreliable internet connections and phone service have created problems with real-time communications, both inside and outside the country.

Deteriorating social, political, and economic conditions have caused many academic researchers to leave Venezuela [28], including the principal investigator and some co-investigators of the MAS. The study has subsequently been managed remotely, resulting in increased administrative complexity and delays. High staff turnover has aggravated the existing difficulty of working in teams. The constant need to hire and train new researchers added complications and negatively impacted morale. New employees struggle with new job duties and procedures under difficult conditions, while remaining employees have to take on increased workloads and responsibilities for training new hires.

#### Increased financial burden

Hyperinflation and currency fluctuation have caused shortages of goods and services in Venezuela [29]. A black market for goods quickly developed, raising the costs of necessities such that average salaries became inadequate. To compensate, minimum wages were increased by the government, almost on a monthly basis, while the official exchange rate was fixed, making it difficult to provide financial support for the staff on the study’s limited budget [30]. The MAS has been further affected by increasing prices of materials needed for research.

#### Political climate - polarized views and mistrust due to foreign funding

Instability caused by the political and economic crisis has led to a continual change in gatekeepers, who influence our access to the population and resources, and to public officials, making it more difficult to establish long-lasting agreements with the public healthcare system. Many participants were also concerned about possible retaliatory measures such as losing their government jobs or losing access to social benefits, as cooperating with U.S.-based scientists could be perceived as some sort of patriotic treason. Relationships with local officials have been always cordial, and we have always presented them with annual summaries and reports. Furthermore, all data are made available whenever any member of the team is asked to participate in a government committee to address a public health issue.

Despite a long tradition of scientific cooperation with the U.S., sanctions imposed in 2014, after a decade of anti-imperialist discourse by the late President Chavez, created yet another challenge. This translated to mistrust related to the U.S. origin of funding awarded by the National Institutes of Health (NIH). Participants became concerned about how their information would be handled by foreign entities, about the commercial value of blood samples, and about the possible perception that participation in the study was anti-government or collaboration with the “enemy.”

As conditions deteriorated, activities and assessments needed to be simplified. During the 2016 follow-up period, the number of laboratory tests had to be restricted, due to scarcity of laboratory reagents. Fuel shortages led us to perform some neuropsychiatric and neuropsychological assessments in the participants’ homes, rather than making them come to our center. Despite these prevailing conditions, MAS participants remain interested in continuing to be in the study for each follow-up evaluation. During the 2013 and 2016 follow-ups, only 33 (5.2%) out of 637 participants contacted declined to participate in the assessments, 52 (8.16%) participants were found dead, and 52 (8.16%) did not complete the battery of assessments. In contrast, 500 subjects successfully completed all study assessments. We do not have an account of how many have emigrated.

For the 2018 follow-up, the community health worker assigned to the catchment area contacted participants only by phone, as fuel shortages were more widespread. This follow-up wave ultimately needed to be postponed, due to damage of the OCT equipment and the difficulty in importing the replacement part in a timely manner. We expected to do this follow-up in 2020, but COVID-19 presented another barrier. Recent contact with participants has been maintained only by phone, but the data collection has been paused for now, due to the pandemic. In addition, the constant need to hire and train new staff has posed a problem for quality assurance. To compensate for the learning curve of new employees, workloads and responsibilities have increased for core employees, impacting the frequency of data quality control. Thus, there was a delay in the identification of errors after data were collected, resulting in a delay in corrective measurements.

### Strategies for mitigating the impact of the humanitarian crisis on research

In retrospect, the challenges that the Venezuelan humanitarian crisis presented to the continuing activities of the MAS seemed to work synergistically, enhancing the impact on different aspects of our operations through **five mechanisms**: 1) **changing community dynamics**, due to changes over time in community structure (access to transportation, electricity, internet, telephone, food, clean water, health services, medications) and composition (paramilitary groups or *colectivos*, black market food distributors or *bachaqueros*, Cuban healthcare providers, selective emigration of professionals); 2) **declining morale of researchers, staff, and participants**, i.e.*,* negative feelings about their situation or dissatisfaction with aspects of the work, family situations, or hardship; 3) **problems with financial viability**, i.e.*,* our ability to meet operating payments and commitments, and to sustain or expand the infrastructure; 4) **components of the research processes**, i.e.*,* our ability to do imaging studies and apply molecular techniques, such as RNA processing; and 5) **health of staff, participants, and their families**, reflecting the impact of poor access to healthcare and medications. Activities implemented to alleviate the challenges have focused on those mechanisms (Table 1). These activities increased as the crisis unfolded.

**Table 1:** Mitigating activities implemented or increased during the humanitarian crisis

Mechanism	Challenges	Activities to counteract the challenges
**Community dynamics**	*Political climate and polarized views *Accelerated deterioration of basic services and safety *Logistics affecting staff and participants *Mistrust in local funding	-Off-site activities (trainings, assessments, events). -Circuits of communication via instant messages/social media. -Scenario exercises. -Liaison with drivers, family members of staff, participants, and law enforcement to facilitate mobilization of participants. -Focus groups, community consultations for solutions.
**Morale of researchers, staff, and participants**	*Ethical issues *Staff emigration *Logistics affecting staff and participants	-Increased communication. -Social marketing through various channels. -Empowering local teams. -Flexible schedules and work from home. -Reflection time included in work hours. -Workshops on coping skills for staff and participants. -Develop sense of ownership in the community. -Weekly debriefing with program managers. -Networking with the emigrated staff.
**Financial viability**	*Augmented burden for administrative and financial management *Mistrust in foreign funding *Supply difficulties	-Adjustment of protected time for research in contracts -Continuous communication with assessment sites. -Direct import of lab supplies and project supplies. -Maintain stock of supplies to use per year. -Efficient negotiations with providers, staff, sites. -Integration with current flows of work. -Organizational capacity development. -Partnership management strategies. -Lobby donors.
**Components of the research processes**	*Logistics affecting staff and researchers *Augmented burden for administrative and financial management *Supply difficulties	-Flexibility, contingency plans. -Standardized training and more supportive monitoring. -Redundant local data storage on hard drives and physical transfer. -Incorporation of home visits and phone interviews for assessments. -Rigorous enforcement of protection of human subjects. -Detailed job descriptions in advertisements. -Weekly documentation of progress.
**Health of staff, participants, and their families**	*Deterioration of basic services and safety *Ethical issues *Staff emigration *Political climate and polarized views	-Free meals provided for participants. -Distribution of free antipyretics and condoms during Zika virus epidemic. -Free medical consultation for participants. -Partnering with private healthcare providers for consultations & exams of participants (neurology, cardiology, psychology, ophthalmology) when needed. -Educational programs. -Management of referral network. -Engagement with local healthcare authorities and directors of healthcare centers.

Many of the **“**Activities to counteract the challenges,” listed in Table 1, were ongoing activities that were successful in supporting the study. As the crisis unfolded, some of those activities were less relevant, and their outcomes were impacted. For example, retention of bilingual and highly qualified personnel has become extraordinarily difficult. We are now more focused on networking with personnel that emigrated and personnel that do not speak English, many of which do not even have a college education.

The ongoing activities of the MAS can be divided into three main categories that provide a broad view of the background and response to the humanitarian crisis, and provide insight into our mitigation approaches for other research projects that might be similarly challenged.

#### Building and sustaining strong research networks

Although the MAS has been housed in the Laboratory of Neurosciences of the University of Zulia, there have always been collaborative interactions with other research groups based in the U.S., Europe, and Latin America. The strategy to sustain these interactions was based on three principles: i) adherence to international ethical and professional standards; ii) sharing of data; and iii) maintenance of social accountability to the population. Having strong supportive research networks positively impacted our ability to understand the changes in community dynamics, and to identify opportunities to keep supporting the community as social changes emerged. One of the most valuable activities was the visits to the community, visiting homes and participating in community assemblies, though this had to be curtailed recently, because of fuel shortages and the pandemic. The direct interaction with international researchers improved the morale of the team during the crisis, particularly as the significance of the efforts and the value of community participation and the whole endeavor of the MAS was reinforced. Troubleshooting sessions led to awareness and acceptance of the limitations, and the need to provide flexible work hours and acquire different skillsets.

As international standards for grants administration were implemented, managerial capabilities were enhanced, and some concepts such as “protected time” were made explicit in subcontracts with local institutions. Uninterrupted funding was secured from different funding agencies based in the U.S. and Europe until 2021. In addition, international partners and the Venezuelan diaspora facilitated access to partnerships that helped support the communities where the MAS had established infrastructure. The international experience of our partners provided flexibility for contingency plans that played a crucial role in the changing conditions of the humanitarian crisis.

#### Enhanced dissemination

Dissemination of research results has been implemented since the beginning of the MAS. We have used a variety of approaches to inform the community and provide opportunities for them to learn about the study and its findings. These approaches include the use of a dedicated community advisory board and social media to facilitate community access to the research, and to promote opportunities for engagement and enrollment in the study. Open houses and tours were hosted to increase understanding of the complexity and value of research, as well as to facilitate dialogue about community needs and interests. The MAS has also organized meetings with local physicians to educate, empower, and motivate their participation in the research activities. Community forums and topical workshops were hosted to educate both the community and researchers. These presentations led to actionable items of interest to the community. An annual research symposium, operating when the political situation in Venezuela was stable, provided an opportunity to present to the community the value of evidence-based healthcare practices. Many of these dissemination activities have been impacted by the humanitarian crisis; however, the bonds already formed with the local population and healthcare personnel have remained largely intact, helping to maintain the study.

#### Capacity building

“Capacity building” refers to interventions that produce sustained change at all levels, from individual to national [31]. Three out of four categories in the framework of Crisp et al., 2000 [32], which characterizes approaches to capacity building, have been actively used by the MAS: (i) bottom-up organizational approaches; (ii) partnerships; and (iii) community organizing approaches [19]. From its initiation, it was clear that the MAS presented an opportunity to support efforts for community improvement and sustainability. We debated extensively about how best to benefit the community and to maximize the role of domestic researchers, to minimize the long-term dependence on international collaborators. We did not want this project to be another instance of “helicopter science,” where foreign investigators descend on a local population, take their blood samples, and leave without contributing any long-term benefit to the local scientists or the population [33]. We held listening sessions, community assemblies, and worked with local non-profit organizations and advocacy groups to discuss how findings from MAS could best be used to benefit and empower the local community. Given the high prevalence of Alzheimer’s disease and other lifestyle-related chronic diseases, like diabetes and hypertension, we tried to provide information and support for disease prevention and mitigation strategies in the community at large, independent of any individual’s participation in our research project. We held several educational programs for the community, as well as for our local scientists, to make sure everyone understood that we saw the MAS as a long-term investment in the community, and that we hoped to leave the situation better than we found it, no matter how the research itself turned out. Our past capacity building activities include:

- The School for Caregivers of Older Adults in Maracaibo [34] and concomitant guide offer education to local caregivers of older adults, with or without dementia, residing at home [35].
- Memory stimulation workshops offer physical, social, and/or cognitive stimulation, as well as a manual of cognitive exercises [22] to be delivered by caregivers or family members.
- More than 50 graduate theses have been produced at the University of Zulia to date. Our local researchers have been invited to join national and international consortia and received awards for the work done within the MAS. At least 20 of our current and former staff have been awarded fellowships and scholarships for presentations at international conferences, to continue their studies, or to pursue further work internationally.

Some of these capacity-building activities have continued during the humanitarian crisis, including the School for Caregivers and the memory stimulation workshops. Others, such as the annual symposium and mass media events have been discontinued, but with the hope and intention to restart them at some future point, when political, economic, and social conditions stabilize.

### Additional strategies for maintaining the MAS

Along with an intense fuel shortage, the COVID-19 pandemic of 2020 has resulted in strict curfews, making continued engagement with participants even more difficult. Personal protective supplies for the healthcare practitioners, such as gloves, facemasks, soap, and hand sanitizer are nearly impossible to obtain. We have continued to adapt our strategies, relying on phone interactions with participants whenever possible and providing protective supplies as part of our research supplies. When telemedicine is not possible, we have deployed in-home assessments to obtain data and to retain participants. Although there is no set strategy that can be applied to all humanitarian crises, the key points for us have been adaptability and proactivity.

Costs of mitigating the impact of the humanitarian crisis on our research have mostly been compensated through partnerships with advocacy groups and humanitarian organizations. We have been funded for research, not humanitarian actions, which could be viewed as a form of compensation or inducement to participate. Although morally justifiable, increased expectations from participants might actually limit the ability to continue the study, if such partnerships are not sustained. Therefore, when we engage in research-related activities, we make them beneficial for the whole community, independently of specific participants.

## Conclusions

Age-related disorders affect millions of people worldwide, including individuals residing in low-and-middle income countries. Unfortunately, older individuals in such countries are likely to be more negatively affected by any humanitarian crises which may arise. The MAS has provided a unique opportunity to study age-related conditions in a Latin American country prior to and during a humanitarian crisis. Despite increasing difficulties, the MAS has continued to: (i) educate community members; (ii) generate unique and extensive data from a large cohort; (iii) conduct high quality scientific research; (iv) provide opportunities for other researchers; and (v) attenuate the national brain-drain by providing employment opportunities for trained professionals.

Most importantly, the MAS has demonstrated that high quality research can be maintained in a developing country with low resources in a time of humanitarian crisis, even though the socio-political crises have slowed down research progress to some extent. We were fortunate to have designed and initiated this longitudinal research project, with a dedicated research team that established a tight bond with the community, prior to the humanitarian crisis in Venezuela. Our established local and international relationships have helped us to overcome deteriorating conditions and increasing barriers to research. Continuation of the MAS is challenging, not only due to the natural debilitation of the older participants, but also to the prolonged humanitarian crisis exacerbated by the COVID-19 pandemic. We continue to formulate research questions that will incorporate the mitigating measures described, as we believe that knowledge obtained in this population is extremely valuable.

## Data Availability

Data sharing is not applicable to this article as no datasets were generated or analyzed during the current study.

## Abbreviations

MAS: Maracaibo Aging Study
OCT: Optical Coherence Tomography
MRI: Magnetic Resonance Imaging
